# Possible stressors in a neonatal intensive care unit at a university hospital

**DOI:** 10.5935/0103-507X.20160041

**Published:** 2016

**Authors:** Kamila Reis Jordão, Lauriane de Assis Proença Pinto, Lucimer Rocha Machado, Laetitia Braga Vasconcellos de Lima Costa, Eduardo Tavares Lima Trajano

**Affiliations:** 1 Faculdade de Fisioterapia, Universidade Severino Sombra - Vassouras (RJ), Brazil.; 2 Faculdade de Enfermagem, Universidade Severino Sombra - Vassouras (RJ), Brazil.; 3 Faculdade de Psicologia, Universidade Severino Sombra - Vassouras (RJ), Brazil.; 4 Laboratory of Biomorfology and Experimental Pathology, Universidade Severino Sombra - Vassouras (RJ), Brazil.

**Keywords:** Intensive care, neonatal, Critical care, Stress, psychological

## Abstract

**Objective:**

To investigate possible stressors to which newborns are exposed in the neonatal intensive care unit.

**Methods:**

The levels of continuous noise were checked by a decibel meter positioned near the ear of the newborn, brightness was observed by a light meter positioned in the incubator in front of the newborn's eyes, and temperature was checked through the incubator display. The evaluations were performed in three periods of the day, with ten measurements taken at one-minute intervals during each shift for the subsequent statistical analysis.

**Results:**

All shifts showed noise above acceptable levels. Morning (p < 0.001), afternoon (p < 0.05) and night (p < 0.001) showed a significant increase compared to the control. The brightness significantly exceeded the normal range (p < 0.01) in the morning. We observed that only one of the incubators was within the normal temperature limits.

**Conclusion:**

The noise, brightness and temperature intensities were not in accordance with regulatory standards and thus might be possible stressors to newborns.

## Abstract (pt)

**Objetivo:**

Verificar possíveis fatores estressantes aos quais os recém-nascidos estão expostos na unidade de terapia intensiva neonatal.

**Métodos:**

Os níveis de ruídos contínuos foram verificados por meio do decibelímetro posicionado próximo ao ouvido do recém-nascido; a luminosidade foi verificada pelo luxímetro posicionado dentro das incubadoras diante dos olhos do recém-nascido; e a verificação da temperatura se deu por meio da visualização do *display* das incubadoras. As avaliações foram realizadas em três períodos do dia, tendo sido realizadas dez medições com intervalo de 1 minuto em cada turno para posteriores análises estatísticas.

**Resultados:**

Todos os turnos apresentaram ruídos acima dos níveis aceitáveis. Manhã (p < 0,001), tarde (p < 0,05) e noite (p < 0,001) apresentaram aumento significativo comparado ao controle. A luminosidade excedeu os padrões de normalidade significativamente (p < 0,01) no período da manhã. Quanto à temperatura, observamos que apenas uma das incubadoras encontrava-se dentro dos padrões de normalidade.

**Conclusão:**

A intensidade dos ruídos, da luminosidade e da temperatura não estavam de acordo com as normas regulatórias, podendo ser assim um possível fator estressante para o recém-nascido.

## INTRODUCTION

Currently, psychological stress is considered to be one of the main factors responsible for changes in the health status of the global population. These changes manifest through physiological, cognitive and behavioral changes and may lead to the emergence of diseases and even death.^(1,2)^ Understanding stress as a psycho-physiological process of the body is important because it allows the diagnosis of responses triggered by the manner in which stimuli are processed.^(3,4)^

The neonatal intensive care unit (NICU) is a stressful environment due to the presence of multiple factors, such as bright light, noise, handlings performed by professionals and little social interaction.^(5,6)^ Thus, despite the high technology and training of the health teams, these factors contribute to changes in the sleep cycle, stress appearance, discomfort and pain.^(7,8)^

A hospital without noise is impossible. However, a hospital with acceptable levels of continuous noise (CN) is a necessary prerogative for the clinical progression of the patient. Therefore, the *Associação Brasileira de Normas Técnicas* (10152/1987) established in-hospital CN values of 35 and 45 decibels (dB) as desirable and acceptable levels, respectively. Furthermore, the American Academy of Pediatrics set specific values by the Consensus Committee on Recommended Standards for Advanced Neonatal Care, which recommends that CN levels should not exceed 45 dB.^(9-11)^

In addition to noise, bright light should also be considered a stressor because it may affect the states of sleep and wakefulness in premature infants and interfere with the entire hormonal circadian rhythm.^(12)^ In the NICU, the use of fluorescent lamps from 40 to 60W/m^2^ is recommended to allow an accurate assessment of the newborn's skin color and provide high visibility during special procedures while at the same time not interfering with the comfort of the newborns.^(7)^ Technical standards related to illumination (NBR 5413) state that the values of artificial illumination in nurseries should not exceed 100 lux.

Body temperature is the result of the balance between the heat production and elimination mechanisms. In newborns (especially in preterm infants), there may be an imbalance between these mechanisms. For full-term babies, the thermoneutral zone in the first hours of life is between 32 and 34°C, but the thermoneutral range varies depending on birth weight and postnatal gestational age and reaches 35 to 37°C for premature newborns with low birth weights in the first days of life.^(7)^ To increase the survival rate of premature newborns, they are placed in closed chambers with the temperature maintained in a specific range, which reduces oxygen consumption and keeps them warm.^(10)^ The same procedure is also suitable for full-term babies who are sick. The incubator heats the atmosphere by convection, providing a warm, stable and uniform environment ranging between 36 and 36.5°C. However, the heated crib emits infrared light that is easily absorbed by the skin, turning it into heat and varying the temperature between 36.5 and 37°C.^(13)^

Reduced exposure to these factors is critical for the good prognosis of the newborn, but often these aspects are neglected by health teams.^(5)^ Thus, the present study evaluated possible stress factors (noise, light and temperature) in the neonatal intensive care unit at a university hospital.

## METHODS

This study was conducted in *Hospital Universitário Sul-Fluminense* (HUSF) and approved by the Research Ethics Committee (No. 1161709) at the *Universidade Severino Sombra*. Parents were requested to sign a free and informed consent form.

The evaluations were performed in three periods [morning (7 am to 9 am), afternoon (13 pm to 15 pm) and night (18 pm to 20 pm)], with 10 measurements taken at one-minute intervals during each 10-minute set. These 10 measurements were only used for the evaluation of lighting and noise. The medical team on shift that day was not informed of the nature of the study in order to avoid any changes in their routine work that could mask the results. The inclusion criterion was to establish that the measurements occurred near the incubators in use at the time of the study.

The measurements were performed with an Instrutherm DEC-460 decibel meter. The equipment was calibrated according to the manufacturer's recommendations with the aid of a standard external acoustic calibrator with a sinusoidal signal of 94 dB at 1 kHz. Then, the device was placed near the ear of the newborn. The decibel meter was set in the compensation circuit "A" and slow response circuit (SLOW) to check the average sound level as indicated for the CN measurements in accordance with regulatory standard NR 15. The reference value recommended by the American Academy of Pediatrics (Consensus Committee on Recommended Standards for Advanced Neonatal Care) and the *Associação Brasileira de Normas Técnicas* NBR 10152/1987 of 45 dB was used as a control. The CN levels were also evaluated during airway aspiration, which is a common procedure in intensive care units.

The measurements were performed with a factory calibrated Light Meter HS1010A. The device was placed in each incubator in front of the eyes of newborns. A control value of 100 lux was used as recommended by the NBR 5413.

The temperature was verified by the display on the incubator because clinicians commonly use this reading to check the temperature during their routine. Normal reference values were taken from those recommended by the Scopes and Ahmed scale, with suggestions for the ideal temperature based on the term range of neutrality as a function of birth weight and postnatal age.^(7)^

### Statistical analysis

The distributions of data obtained from the sound level meter and light meter were verified by the Kolmogorov-Smirnov test. Then, one-way analysis of variance (ANOVA) with the Bonferroni post hoc test was used to compare the means with GraphPad Prism 5^®^. The level of significance was set at p < 0.05, and the results are expressed as the mean ± standard error of the mean. For temperature, we only tested whether the identified values fit the Scopes and Ahmed scale.

## RESULTS

**Figure 1:**
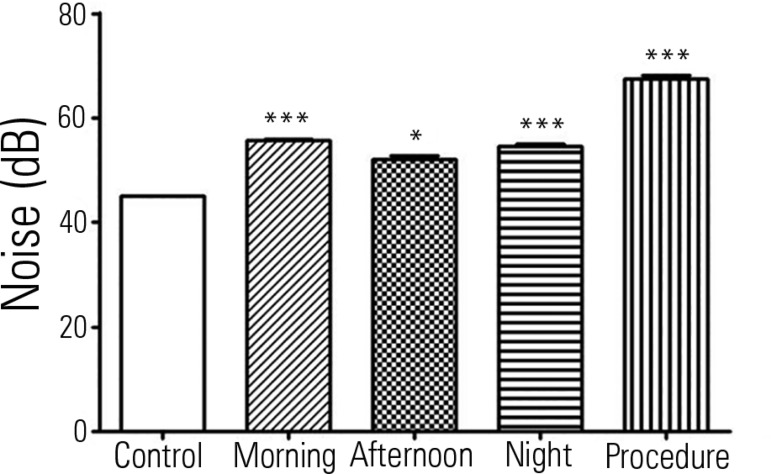
Quantification of continuous noise in the neonatal intensive care unit. Data presented as the mean ± standard error of the mean. * Significant difference with p < 0.05; *** Difference p < 0.001; both compared to the control. We used 45 dB as the reference value, which is recommended by the American Academy of Pediatrics and *Associação Brasileira de Normas Técnicas* NRB 10152/1987.

All shifts had average CN levels above the acceptable limit. The levels during the morning (23.48%; p <0.001), afternoon (15.7%; p <0.05) and night (21%; p <0.001) shifts were higher than the recommendations. Additionally, we observed an increase of 49.7% (p <0.001) in CN while performing a routine procedure (aspiration) compared with the control (Figure 1).

**Figure 2:**
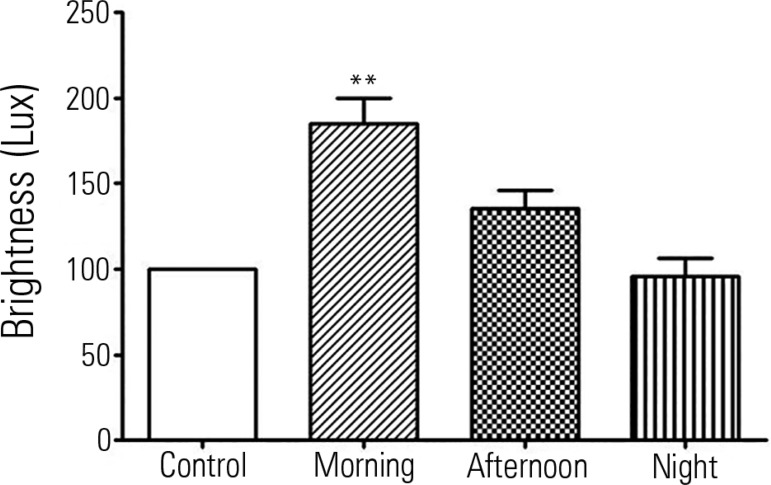
Quantification of illuminance in the neonatal intensive care unit. Data presented as the mean ± standard error of the mean. ** Significant difference with p < 0.01 compared to the control. For the control, we used 100 lux, the amount recommended by the *Associação Brasileira de Normas Técnicas* NBR 5413.

Regarding brightness, we found that the illuminance during the morning shift was 84.8% above the recommended limit (p < 0.01). Significant differences were not observed in the other shifts (Figure 2).

Only one of the incubators in use at the time of the study was within the normal temperature limits in all three periods (Table 1).

**Table 1:** Temperature of the neonatal intensive care unit incubators

Weeks of life/hour	Reference temperature (ºC)	Temperature (morning) (ºC)	Temperature (afternoon) (ºC)	Temperature (night) (ºC)
12 - 24 hours	31.0 - 35.4	30.5	31.2	31.2
2 to 3 weeks	30.5 - 33.6	28.9	29.8	29.9
3 to 4 weeks	30.5 - 33.0	30.5	31.2	31.
4 to 5 weeks	29.5 - 32.3	---	---	---
5 to 6 weeks	29.0 - 31.8	31.6	32.7	32.5

The temperature values are shown as the average using the normal Scopes and Ahmed scale as the reference.^(14)^

## DISCUSSION

The NICU is an aggressive environment that is impersonal and difficult to adapt. This environment is full of intense and constant light, noise, temperature changes and disruption of the sleep cycle due to repeated assessments and procedures that occur more or less frequently depending on the severity of the newborn.^(11)^

Fasolo et al. conducted a study that found that 33.3% of the assessed incubators were 60 dB above the recommended tolerable levels. Moreover, they observed that the average non-CN generated by manipulation of the incubator access ports ranged from 96.2 dB (opening) to 107 dB (closing). Fasolo et al.^(13)^ observed that the intensity of CN might vary according to shift.^(15)^ Heidemann et al. conducted a study in a coronary care unit and observed a positive correlation between CN and stressors.^(16)^ Sampaio Neto et al. investigated the perception of professionals working in the ICU regarding noise and found that 97.3% reported that the unit had moderate to intense noise.^(17)^ This study identified CN as above acceptable levels in different shifts.

Consensus 2008 reports that keeping premature infants in continuous shade deprives them of circadian information, which should be received by the end of pregnancy. Periodic low intensity brightness (180 to 200 lux) stimulates the development of the biological clock, which becomes an increasingly important part of neonatal care. Thus, not only can excessive exposure to light negatively affect development but continuous shade is also not indicated.^(18)^ Souza et al. conducted a study that found that the artificial lights in the neonatal ICU remained on all the time.^(19)^ There was no possibility of reducing brightness at certain times of the day, and the only procedure to reduce brightness involved placing a sheet over the incubator (called a "little roof"). The present study found a significant increase in brightness in the morning (p < 0.01). Additionally, the unit used fabrics (light blockers), which were placed over the incubators continuously. We observed that there was no eye protection for newborns in heated cribs.

According to Martins et al., the temperature of the incubator should be kept in the thermoneutral zone to regularly control the body temperature of the newborn, who will be exposed to relative humidity of 85% in the first week of life. From the third week of life, the relative humidity should be gradually reduced to 60% and maintained until the preterm reaches a weight of 1500 grams.^(20)^ This study found that most of the incubators were not in accordance with the recommended standards.

Terenan et al. investigated the possible correlation between the quality of life and the severity of diseases through the Medical Outcomes Study 36 (SF-36) and Acute Physiology and Chronic Health Disease Classification System II (APACHE II) questionnaires, respectively, in patients within 72 hours of ICU admission. Despite the study limitations noted by the authors, the study showed a weak correlation between quality of life and severity of disease.^(21)^ Vesz et al. investigated functional and psychological aspects immediately after ICU discharge using the modified Barthel scale and a hospital anxiety and stress questionnaire and observed a high incidence of depressive symptoms, anxiety and sleep disorders.^(22)^

## CONCLUSION

The continuous noise, light and temperature intensities were not in accordance with regulatory standards and might be stressors for newborns. Special attention is needed regarding the stressors in this environment by the medical team to avoid possible psychophysiological changes that adversely interfere with the newborn's prognosis.
